# Cases of Poisoning by Chloride of Zinc

**Published:** 1849-12

**Authors:** 


*Cases of Poisoning by Chloride of Zinc.*—Dr. Thomas Stratton, R. N., relates in the Edinburg Medical and Surgical Journal, for October, 1848, two cases of poisoning with chloride of zinc. In both cases a wine-glassful of solution was swallowed, containing in one case, about twelve grains of the salt, and in the other, about two hundred grains. In the latter case, burning pain in the gullet, burning and griping pain in the stomach, great nausea, and sense of coldness, were instantly felt. Vomiting followed in a few minutes. Dr. Stratton saw this patient twenty minutes after the accident, and instantly made a strong solution of home-made brownish soap, of which he made the patient swallow at intervals, three or four pints. Afterwards, olive oil was given, and the patient recovered. The other case was not seen by any medical man; but it also terminated favorably. Dr. Stratton suggests either soap, or carbonate of soda or of potash, as antidotes of chloride of zinc, and supports his suggestion by the recital of experiments which he has performed.—*London Jour. of Med.*

